# Effector-specific motor simulation supplements core action recognition processes in adverse conditions

**DOI:** 10.1093/scan/nsad046

**Published:** 2023-09-09

**Authors:** Gilles Vannuscorps, Alfonso Caramazza

**Affiliations:** Psychological Sciences Research Institute, Université catholique de Louvain, Place Cardinal Mercier 10, 1348, Louvain-la-Neuve, Belgium; Institute of Neuroscience, Université catholique de Louvain, Avenue E. Mounier 53, Brussels 1200, Belgium; Department of Psychology, Harvard University, Kirkland Street 33, Cambridge, MA 02138, USA; Department of Psychology, Harvard University, Kirkland Street 33, Cambridge, MA 02138, USA; CIMEC (Center for Mind-Brain Sciences), University of Trento, Via delle Regole 101, Mattarello TN 38123, Italy

**Keywords:** action recognition, working memory, motor simulation

## Abstract

Observing other people acting activates imitative motor plans in the observer. Whether, and if so when and how, such ‘effector-specific motor simulation’ contributes to action recognition remains unclear. We report that individuals born without upper limbs (IDs)—who cannot covertly imitate upper-limb movements—are significantly less accurate at recognizing degraded (but not intact) upper-limb than lower-limb actions (i.e. point-light animations). This finding emphasizes the need to reframe the current controversy regarding the role of effector-specific motor simulation in action recognition: instead of focusing on the dichotomy between motor and non-motor theories, the field would benefit from new hypotheses specifying when and how effector-specific motor simulation may supplement core action recognition processes to accommodate the full variety of action stimuli that humans can recognize.

## Introduction

Every day, we see people executing sequences of body movements under a wide range of illumination, viewpoints and occlusion in more or less cluttered and crowded environments. Despite the challenges imposed by these complicating factors, most of the time, we recognize what they are doing, e.g. that they are ‘texting while walking’, ‘smiling’ or ‘running’ (e.g. Ikeda *et al.*, 2013; Ikeda and Watanabe, 2016; Fademrecht *et al.*, 2017). By action recognition, we refer to the ability to categorize observed body movements and postures as a specific instance of a known category of action—to ‘recognize’ that the observed body movements constitute an instance of the action ‘doing a cartwheel’, for instance. Studies exploring the cognitive and neural bases of this ability have consistently reported that observing other people performing actions activates effector-specific imitative motor plans in the observer’s mind/brain (Buccino *et al*., 2001; Caspers *et al*., 2010; Cracco *et al.*, 2018; Fadiga *et al.*, 1995). However, whether, and if so when and how, such effector-specific ‘motor simulation’ contributes to action recognition remains unclear.

The most influential early motor simulation theories attributed a critical role to effector-specific motor simulation in action recognition (Blakemore and Decety, 2001; Jeannerod, 2001; Rizzolatti *et al.*, 2001; Rizzolatti and Craighero, 2004). The most popular of these theories is the direct-matching hypothesis, proposed by Rizzolatti *and colleagues* following the discovery of mirror neurons in the macaque monkey’s brain (Rizzolatti *et al.*, 2001). Based on the properties of these neurons, the authors proposed that they constitute a matching system that automatically translates the results of the visual analysis of observed body movements into their corresponding motor commands in the observer’s brain. Mirror neurons would allow action recognition because the observer ‘knows’ what s/he is doing when s/he performs the same action (but see Csibra, 2007 for critical discussion). Thus, on this view, although actions may be recognized purely visually (without effector-specific motor simulation), direct visuomotor matching is the only mechanism ‘by which the meaning of the acts that are being observed are understood immediately’ (Rizzolatti and Sinigaglia, 2008, p. 136) without the need of ‘additional complex inference processes’ (Rizzolatti *et al.*, 2014; Giese and Rizzolatti, 2015) and by which the observer is able to understand an observed action at a level that goes beyond its mere visual features (Nelissen *et al.*, 2005; Rizzolatti and Sinigaglia, 2010). Accordingly, Rizzolatti and Sinigaglia (2010, 2016) refer to the finding that some brain-damaged individuals with motor circuit lesions become unable to recognize pictures or video clips of familiar actions as a ‘compelling argument for the crucial role of mirror neurons in this function’.

It has since become clear that such strong versions of the motor simulation theories, in which effector-specific motor simulation is necessary for efficient action recognition, are untenable. Many studies have reported action recognition difficulties in patients suffering from brain damage involving different parts of the motor system in the context of different etiologies: patients with apraxia (a disorder affecting the capacity to perform actions despite preserved basic motor and somatosensory functions), motor-neuron disease, Parkinson’s disease and cortico-basal degeneration present with difficulty to recognize action pictures and pantomimes (Buxbaum *et al.*, 2005; Cotelli *et al.*, 2006; Negri *et al.*, 2007; Silveri and Ciccarelli, 2007; Grossman *et al.*, 2008; Pazzaglia *et al.*, 2008a, 2008b; Papeo *et al.*, 2010). However, the reported action recognition impairments cannot unambiguously be ascribed specifically to motor system damage because, in these disorders, brain lesions generally extend outside the motor system and most patients also present with other cognitive difficulties, such as executive function, attentional and/or visuospatial disorders. Furthermore, at odds with the prediction of the strong versions of the motor simulation theories, there are also many reports of brain-damaged patients who, despite impaired action production, achieve normal performance in naming, or matching-to-a-word, pictures, video clips and pantomimes of actions (Negri *et al.*, 2007; Kalénine *et al.*, 2010; Papeo *et al.*, 2010). In a 3-year longitudinal study, for instance, patient J.R. presented with increasing action production difficulty resulting from progressive bilateral atrophy in cortical and subcortical regions involved in the sensorimotor control of actions, notably the superior parietal cortex, the primary motor and premotor cortex, the inferior frontal gyrus and the basal ganglia. Despite the extensive damage to these structures, which are assumed to underlie motor simulation, J.R.’s ability to recognize actions remained intact and comparable to that of control participants in both accuracy and speed (Vannuscorps *et al.*, 2016).

The hypothesis that action recognition is mediated by effector-specific motor simulation is also difficult to reconcile with reports of typically efficient action recognition in individuals who cannot rely on such mediation because of congenitally absent or paralyzed limbs (Vannuscorps *et al.*, 2020a; Vannuscorps *et al.*, 2020b; Vannuscorps and Caramazza, 2016b). Action recognition through direct matching is a two-step process: (i) observed body movements are translated into the corresponding effector-specific motor commands in the observer’s brain and (ii) the simulation allows observers to ‘retrieve’ information about what action these movements allow them to produce when they carry them out. In this scenario, previous motor experience with observed body movements is critical for effector-specific motor simulation to occur, and action recognition efficiency is assumed to depend on the similarity between the observed movements and those produced by the viewer (Calvo-Merino *et al.*, 2006; Swaminathan *et al.*, 2013; Turella *et al.*, 2013; Tye-Murray *et al.*, 2013). These two steps in action recognition are not available to observers with congenitally absent or paralyzed limbs. Extant evidence suggests that the motor cortex does not contain representations of congenitally absent or paralyzed limbs (Reilly and Sirigu, 2011; Striem-Amit *et al.*, 2018). Rather, the specific parts of the somatosensory and motor cortices that would normally represent the ‘absent’ or paralyzed limbs are allocated to the representation of adjacent body parts (Kaas *et al.*, 1983; Funk *et al.*, 2008; Stoeckel *et al.*, 2009; Makin *et al.*, 2015; Striem-Amit *et al.*, 2018). In addition, and in any event, individuals with congenitally missing or paralyzed limbs have obviously never themselves executed any action using these missing or paralyzed limbs. Nevertheless, individuals born without upper limbs have been shown to be as fast and accurate as control participants at recognizing pictures and video clips of upper-limb actions (Vannuscorps *et al.*, 2013), and the quantitative and qualitative performance of some individuals born with facial paralysis is indistinguishable from that of control participants in challenging lip-reading and facial expression recognition tasks (Vannuscorps *et al*., 2020b; Vannuscorps *et al.*, 2020a).

**Fig. 1.**
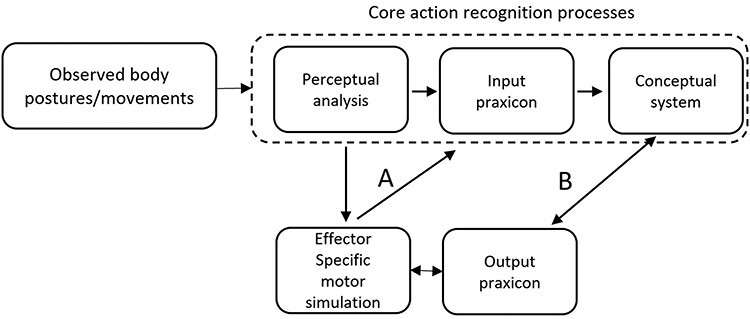
The schematic representation of three different ‘routes’ to action recognition. Core action recognition refers to the automatic, effortless matching of familiar body postures and movements onto a corresponding stored action representation. When core action recognition fails, action recognition may be supplemented by effector-specific motor simulation (see text for detail).

These findings challenge the central premise of effector-specific motor simulation theories: they demonstrate that it is possible to account for efficient action recognition without effector-specific motor simulation. Instead, they support theories of action recognition according to which action recognition results from a matching of observed body postures and movements to mental representations (descriptions) of the body postures and movements that characterize known actions stored in memory (Gonzalez Rothi *et al.*, 1991; Giese and Poggio, 2003; see Figure 1).

In the study of language, the term ‘input lexicon’ is used to describe components of the language system that store abstract information about words that one has previously read (‘input orthographical lexicon’) or heard (‘input phonological lexicon’). This information is deemed ‘abstract’ in the sense that it is invariant to low-level sensory features. For instance, at this stage of processing, a large capital letter ‘M’ printed in red ink in Times New Roman refers to the same ‘abstract’ representation of the letter ‘M’ as a lowercase ‘m’ printed in green in Arial (Grainger *et al.*, 2008). By analogy, in the field of action recognition, Heilman and Rothi (1993) used the term ‘input praxicon’ to refer to abstract representations of known actions stored in memory. Although what exactly is represented in the input praxicon remains insufficiently articulated, representations of known actions likely include information about a series of ‘units’ of body postures and movements that together make up human actions. This information likely includes what are the parts of the body involved (*vs* those whose posture and kinematic are not relevant) and for these body parts their characteristic configuration (e.g. fingers stretched and elbow flexed), position, orientation and kinematic (orientation, amplitude, number of repetitions, speed, velocity).

When one sees an action, it may be recognized in three stages. First, a visuo-perceptual analysis of that action decomposes it into its constituent postural and kinematic units (Giese and Poggio, 2003; Csibra, 2007). In the field of object recognition, such initial parsing of the visual scene, referred to as ‘mid-level’ vision, is assumed to compute a series of visual features such as, for example, the objects’ elongation axis, left and curvature (Palmer and Rock, 1994; Sekuler, 1996; Ungerleider and Bell, 2011; Vannuscorps *et al.*, 2021). When one sees an action, the set of ‘units’ computed is likely to correspond to the set of representational units that characterize known actions in the input praxicon. The result of this operation is compared with representations of known actions stored in the input praxicon. If a reasonably good match is found, the observed action is automatically, rapidly, effortlessly ‘recognized’ as an instance of a known category of actions (e.g. as an instance of ‘waving goodbye’). Once recognized as an action of a certain type, access to a conceptual system allows the observer to retrieve knowledge about the action, like its typical cause, purpose and results, the typical agent and instrument involved, the needed force, approximate duration and so on. Following a similar proposal in the field of object recognition (DiCarlo and Cox, 2007), we refer to the rapid, effortless, automatic recognition of actions that results from these three stages as ‘core action recognition’.

On this view, the observation of body movements is accompanied by two distinct types of ‘motor simulation’ in the service of imitation, emulation and motor learning (Heilman and Rothi, 1993; Buccino *et al.*, 2004). Once analyzed as a set of relevant gestural ‘units’, observed body movements and/or postures are automatically translated into the motor commands that the observer would use to execute the same body postures and/or movements (e.g. Brass and Heyes, 2005). This ‘gesture-to-gesture’ or ‘effector-specific’ imitation/simulation must be distinguished from ‘action-to-gesture’ or ‘effector-independent’ emulation/motor simulation, which results from action recognition. Once conceptual knowledge has been accessed, it activates associated representations such as the phonological and orthographic lexicons that encode the spoken and written forms associated with the action (to name it; Shelton and Caramazza, 1999) and the ‘output praxicon’ that stores learned motor programs associated with action execution and object use (to execute it; Heilman and Rothi, 1993). In other words, once observed body movements have been recognized as an act of a particular type, the motor programs that the observer would use to execute that action are automatically evoked. The automatic activation of post-conceptual representations is supported by a range of findings, such as the demonstration that pictures of objects automatically activate their related phonological (Navarrete and Costa, 2005; Meyer and Damian, 2007) and motoric (Bub and Masson, 2006) content and that observers born without upper limbs activate brain regions involved in the execution of mouth and lower-limb actions when they observe hand action (Gazzola *et al.*, 2007; Aziz-Zadeh *et al.*, 2012).

However, evidence that efficient action recognition is possible without effector-specific motor simulation does not imply that action recognition may not, in some conditions, require additional processing resources. Indeed, the idea that the core cognitive and neural mechanisms underpinning a given function may be supplemented by other ones when tasks or stimuli become more challenging is widely accepted. Although not necessary for auditory speech perception, for instance, it is well documented that lip-reading enhances speech perception under difficult listening conditions (Akeroyd, 2008; Rönnberg *et al.*, 2013). Although object recognition may be largely solved by feedforward visual processing, additional recurrent processes become necessary under challenging conditions (Tang *et al.*, 2014, 2018).

Core action recognition is, by design, limited to cases in which the result of the visuo-perceptual analysis of the actors’ body posture and movements may be matched onto a corresponding memory trace. When this is not possible, one must necessarily rely on additional resources and mechanisms. In line with this, for instance, information about the context in which an action takes place (e.g. the room) has been shown to facilitate specifically the recognition of actions that are unfamiliar (Wurm *et al.*, 2017) or made perceptually ambiguous (Wurm and Schubotz, 2017).

As discussed earlier, ‘action-to-gesture’ ‘effector-independent’ motor simulation results from, and therefore cannot contribute to, action recognition (Gazzola *et al.*, 2007). However, we see at least two ways by which effector-specific motor simulation may support the recognition of actions when core action recognition fails (see Figure 1). Previous evidence has shown that the ability to maintain meaningless, uninterpreted body movements and postures in memory, even for just a few seconds, is augmented by effector-specific motor simulation (Moreau, 2013; Gao *et al.*, 2015; Galvez-Pol *et al.*, 2020). Individuals born without upper limbs have been shown to be significantly less good than typically developed participants at maintaining hand postures in memory even for a few seconds (Vannuscorps and Caramazza, 2016a). In cases such as these, when core action recognition fails, one way in which effector-specific motor simulation may contribute to action recognition is by allowing the rehearsal of the observed gestures/postures, extending the time they will be available (in a motor format) for the more effortful recognition process. During this time window, the rapid decay of the initial transient visual trace is likely to transform action recognition from a process of matching a visual input onto a representation stored in memory to a process of attempting to identify which known action corresponds to the covertly executed movements (Figure 1A). In other words, although action recognition may operate rapidly on a pure visuo-perceptual basis when an action is familiar and perceived in optimal viewing conditions, motor simulation may offer to any observer able to covertly imitate the observed gestures a useful ‘tool’ to extend the processing time window available to interpret actions when stimuli are more difficult to match rapidly onto memory representations—a role akin to that attributed to the articulatory loop component of working memory (Baddeley, 2012) in the ability to recognize speech under adverse conditions (Rönnberg *et al.*, 2010).

During this time window, effector-specific motor simulation may also contribute by activating corresponding actions (if any) in the observer’s motor repertoire or ‘output praxicon’ (Heilman and Rothi, 1993; Figure 1B). This contribution would be akin to the one described by Rizzolatti and Sinigaglia (2010): ‘…observing actions performed by another individual elicits a motor activation in the brain of the observer similar to that which occurs when the observer plans their own actions, and the similarity between these two activations allows the observer to understand the actions of others without needing inferential processing’ (p. 268). As such, this process would be similar to that available to brain-damaged patients with pure alexia, who are unable to match perceptual representations of letters with their stored representations but may circumvent this disorder by tracing the shape of the letters (overtly or covertly) and, thereby, recognize the letter because they know what letter they trace when they typically use these motor programs (Kashiwagi and Kashiwagi, 1989; Lott *et al.*, 2010; Starrfelt *et al.*, 2013).

In a previous study (Vannuscorps and Caramazza, 2016b), we addressed this possibility by comparing the ability of five individuals born without upper limbs and matched control groups of typically developed participants to recognize upper-limb actions presented in either a familiar or an unfamiliar format. In a first experiment, we showed them video clips of an actress executing pantomimes of various familiar upper-limb actions (e.g. playing the guitar and cutting with scissors) and used a gradual unmasking paradigm to measure the quantity of information (i.e. number of frames) necessary for the participants to recognize these pantomimes. By showing familiar actions in a familiar format, we aimed at assessing the efficiency of participants’ core action recognition system. To test the hypothesis that effector-specific motor simulation may enhance action recognition when core action recognition fails, we also needed stimuli that were sufficiently dissimilar from stored representations of known actions to hamper their automatic, rapid recognition by visuo-perceptual matching. Therefore, in a second experiment, we also measured their ability to recognize point-light animations (PLAs) of upper-limb (e.g. playing the violin) and lower-limb (e.g. moonwalking) actions. PLAs of actions are extremely impoverished stimuli in which the stimulus is reduced to only a few dots depicting the spatiotemporal location of the major joints of the actor’s body. Participants had never experienced PLAs before, and these stimuli are very different from natural stimuli. Therefore, we assumed that these stimuli could benefit from effector-specific motor simulation.

The results of the first experiment were clear-cut: all five IDs performed as well as the control participants. The results of the second experiment were mixed, however. In line with the possibility that effector-specific motor simulation may contribute to action recognition when core action recognition fails, three IDs recognized significantly fewer upper-limb than lower-limb actions (in comparison to the controls). However, two others were slightly better at recognizing PLAs of upper-limb than lower-limb actions in comparison to the controls. We concluded that the performance of these two individuals ‘…demonstrate(s) the ability of the visuo-perceptual system, in the absence of motor simulation, […] to perceive and interpret observed actions efficiently even when they are presented in extreme, impoverished conditions’ (Vannuscorps and Caramazza, 2016b, p. 89).

We have since found a reason to moderate and clarify this conclusion. A reanalysis of the performance of the two IDs who performed slightly better for manual than non-manual actions by means of the Bayesian Standardized Difference Test (Crawford and Garthwaite, 2007) indicated that this advantage was nevertheless smaller than that of ∼30% of the typically developed population. This left a narrow space for a role of motor experience in the ability to recognize actions in adverse conditions. This reconsideration of the original results led to the follow-up study reported herein. The goal was to gain more power to detect an impact of motor simulation on action recognition, if any, by testing additional individuals born without upper limbs. The results of the new analyses led us to reconsider our previous conclusions. In line with the conclusion of our previous study, we found conclusive evidence that the IDs recognize upper-limb actions presented as pantomimes as efficiently as typically developed individuals. However, and in contrast with the conclusion of the previous study, we also found that the IDs, as a group, are significantly less accurate at recognizing PLAs of upper-limb than lower-limb actions.

## Material and methods

The study was approved by the biomedical ethics committee of the Cliniques Universitaires Saint-Luc, Brussels, Belgium, and all participants gave written informed consent prior to the study.

### Participants

We report here the results of two individuals born without upper limbs (ID6–7) and of one individual born without upper or lower limbs (ID8) in addition to the five IDs (ID1–5) already reported in our previous study (Vannuscorps and Caramazza, 2016b) and compared the performance of this group (four females and four males; mean age ± s.d.: 48 ± 12) to that of the 27 typically developed age-matched control participants reported in the original study (all right-handed, 17 females and 10 males; mean age ±  s.d.: 45 ± 10) without any history of psychiatric or neurological disorder. Information about the IDs’ body schema was obtained through visual examination and interview. Information about prosthetic and phantom limb history was obtained through a questionnaire and complemented by interview when necessary. None of the IDs reported any history of phantom limb sensation. All the IDs had typical lower limbs, except ID4 who had a shortened right leg, and ID8, who had missing lower limbs. Other relevant information is summarized in Table 1.

**Table 1.** Demographic and clinical data of the individuals born without upper limbs

ID	Sex	Age (years)	Education (years)	Right upper limb	Left upper limb
ID1	M	52	5	±12 cm humerus or ulna directly fused to a hand composed of fingers 1 and 3	Aplasia
ID2	F	47	3	Shortened arms (±30 cm) directly fused to the hands and oligodactyly of the hands (right hand: digits 1, 3 and a shortened thumb with two hypoplastic phalanges; left side: digits 1, 4 and a ±1 cm rudimentary thumb with two hypoplastic phalangeal-like bones)	
ID3	F	49	0	Anatomically and functionally typical hands positioned a few centimeters below the shoulders (no arms or forearms)	
ID4	F	27	4	Aplasia	Aplasia
ID5	M	37	5	Aplasia	Shortened (±15 cm) arm, no forearm and no hand
ID6	M	60	2	Aplasia	Aplasia
ID7	M	58	3	Aplasia	Aplasia
ID8	F	60	3	Two rigid fingers attached to a ±15 cm segment below the shoulder	

a Participants’ number of years of higher education at the time of testing.

Note that we also recruited and tested another individual born without upper or lower limbs in the context of this study (ID9). We decided against reporting his results because he could not be tested in the same conditions as the other participants. During testing, ID9 was lying face down next to the 5.5-inch smartphone screen on which he performed the experiment. Therefore, the size of the stimuli had to be severely reduced.

### Stimuli and procedure

All participants performed three experiments. The first two were identical to those reported in the original article (Experiments 1 and 2 in Vannuscorps and Caramazza, 2016b). In Experiment 1, participants viewed video clips of an actress pantomiming 20 different upper-limb instrumental actions (e.g. playing a guitar; Agostini *et al.*, 2019). Only body movements were shown, without any object or context. In most of the stimuli (*n* = 16), only the upper limb(s) moved (the face remained neutral and the body did not move). In the four other stimuli (to shoot a basketball, to play golf, to throw a ball and to shoot a bow and arrow), the upper-limb movements were accompanied by coarse movements of the body and shoulders. All video clips were sized 978 × 550 pixels and had 30 frames/seconds. From each original movie, we created 14 clips in which the number of frames ranged from 10 (330 ms) to 75 (2640 ms) in steps of five (165 ms). During the experiment, participants viewed the 14 versions of each video in a row, from the shortest to the longest one. The size of the actress on the computer screen was ∼10 cm. Each trial began with the presentation of a black screen for 1000 ms, followed by the video clip and a screen on which was written the question ‘What was the action mimed by the actress?’. Participants responded orally to the question and the experimenter wrote down their responses. They were encouraged to provide a response at each step, even if they were not sure. There was no time constraint for responding, but participants were asked not to respond before the end of each video clip. An item was scored correct at a given level of demasking (from 1 to 14) if it was identified correctly also at all subsequent levels and was scored 15 if not recognized.

In Experiment 2 (Experiment 2 in Vannuscorps and Caramazza, 2016b), participants viewed video clips depicting an actor reduced to 12 light dots (∼5 mm diameter) corresponding to captors originally placed on his main joints (left of the head, shoulders, elbows, wrists, left of pelvis, knees and ankles; ‘PLA’) executing 20 upper-limb (e.g. fishing) and 20 non-upper-limb actions (e.g. walking backward). The video clips were sized 700 × 1024 pixels, had 33 frames/seconds and lasted 5 s. These stimuli were chosen from a set of 83 PLAs of actions created from a motion capture database (asf/amc format obtained from the Carnegie Mellon University Motion Capture Database) with a software developed locally. These 83 point-light displays were shown to two groups of 20 control subjects. Participants of Group 1 (mean age = 24.8; 10 males) were presented PLAs and asked to name them. Participants of Group 2 (mean age= 23; five males) were presented the same PLAs as Group 1 except that the upper limbs were masked (elbows and wrist removed) in order to determine the role of these limbs in the identification of each action. From this preliminary study, 20 upper-limb actions (Group 1’s mean = 87.75% and Group 2’s mean = 8.25%) and 20 lower-limb actions (Group 1’s mean = 93.75% and Group 2’s mean = 92%) were selected. During the experiment, participants viewed the 40 video clips in the same pseudo-randomized order. The size of the actor on the computer screen was ∼9 cm. Each trial began with the presentation of a black screen for 1000 ms, followed by the video clip and a screen on which was written the question ‘What was the action?’. Participants responded orally to the question, and the experimenter wrote down their responses. They were encouraged to provide a response for each stimulus, even if they were not sure. There was no time constraint for responding, but participants were asked not to respond before the end of each video clip.

In Experiment 3, participants viewed video clips of an actor pantomiming the 20 upper-limb actions depicted as PLAs in Experiment 2. Only body movements were shown, without any object or context. All video clips were sized 720 × 1280 pixels, had 25 frames/seconds and lasted 5 s. During the experiment, the size of the actor on the computer screen was ∼7 cm. Each trial began with the presentation of a gray screen for 1000 ms, followed by the video clip and a screen on which was written the question ‘What was the action mimed by the actor?’. Participants responded orally to the question and the experimenter wrote down their responses. They were encouraged to provide a response, even if they were not sure. There was no time constraint for responding, but participants were asked not to respond before the end of each video clip.

During the experiments, the control participants and IDs 1–5 were seated in front of a computer screen located at a distance of about 60 cm. The experiments were controlled with the E-Prime 2.0 software (Psychology Software, 2016, Pittsburgh, PA) and presented on a 15.6-inch Dell Latitude E5530 anti-glare laptop screen set at 1366 × 768 pixels and 60 Hz. IDs 6–8 were tested remotely under the supervision of the experimenter through a visual conference system. The experiments were controlled by the online testable.org interface (http://www.testable.org), which allows precise spatiotemporal control of online experiments. At the beginning of each experiment, IDs 6–8 were sitting in front of a computer screen and instructed to set the browsing window of the computer to full screen and minimize possible distractions (e.g. TV, phone, etc.). Next, a calibration procedure ascertained a homogeneous presentation size and time on all computer screens. Finally, the participants started the experiment.

## Results

**Fig. 2.**
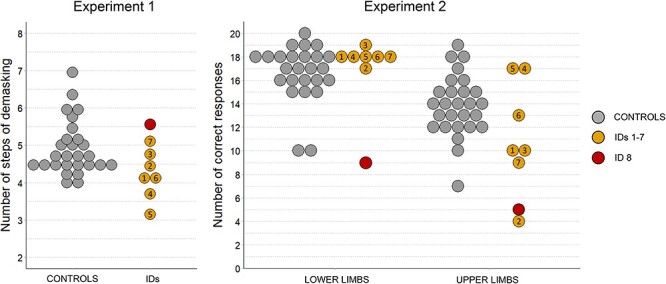
Results of Experiments 1 and 2 by individual participant and group.

The results of Experiments 1 and 2 are displayed in Figure 2. We first analyzed the data of Experiment 1 and tested whether the IDs’ (1–8) lack of motor experience with the upper limbs impacted their ability to recognize manual actions depicted as pantomimes. This was not the case. Descriptive analysis of the data indicated that the IDs (median = 4.3) needed on average fewer steps of demasking than the controls (median = 4.7) and the results of a Mann–Whitney *U* test provided no support for the hypothesis that the IDs are less efficient than the controls at identifying pantomimes of actions (W = 147.5, *P* = 0.94). An additional Bayesian Mann–Whitney *U* test performed with a Cauchy prior lefted around zero and with a width parameter of *r* = 0.707 for effect size on the alternative hypothesis indicated that the data were 6.02 times (Bayes factor) more likely under the hypothesis that IDs are better or as good as the controls at identifying pantomimes of actions than under the hypothesis that the IDs are less efficient.

Then, we analyzed the data of the PLA naming task. We first tested whether the IDs 1–7, as a group, were less accurate at recognizing upper-limb than lower-limb actions, everything else being equal—that is, in comparison to the baseline provided by the performance of the typically developed controls for both action categories. Descriptive analysis of the data indicated that there was indeed a larger difference between manual and non-manual actions in the IDs (medians = 10 and 18, respectively) than in the controls (medians = 14 and 17, respectively). The results of an analysis of variance (ANOVA) with group as between-subject factor and effector as within-subject factor on the data transformed by an Adjusted Rank Transform (ART) procedure (Leys and Schumann, 2010) indicated that this interaction between group and effector was significant (*F* (1, 32) = 8.08, *P* = 0.008, η^2^ = 0.2). The results of an additional Bayesian repeated-measure ANOVA indicated that the model including an interaction between group and effector was 6.8 times (Bayes factor) more likely than the model including only the main effects. Second, we tested whether ID8, who is missing both upper and lower limbs, was less accurate than control participants for both lower- and upper-limb actions. The results of two modified *t*-tests (Crawford and Howell, 1998) indicated that this was the case (both modified *t*s <−3.23, both *P*s < 0.002). Finally, we tested whether IDs 1–8, as a group, were less accurate at recognizing PLAs of upper-limb actions than the typically developed controls. The results of both frequentist (W = 155, *P* = 0.03) and Bayesian (same parameters as abovementioned for the alternative; Bayes Factor = 1.6) Mann–Whitney *U* tests supported this hypothesis over the alternatives.

Third, we analyzed the data from Experiment 3 and tested whether the IDs’ (1–8) lack of motor experience with the upper limbs impacted their ability to recognize pantomimes of the upper-limb actions depicted as PLAs in Experiment 2. The goal was to ensure that the difficulty to recognize upper-limb actions detected in Experiment 2 could be attributed to the format of the stimuli (PLAs), rather than to the manual actions being less familiar or harder to recognize than in Experiment 1. In line with this, both the IDs (mean = 19.89, s.d. = 0.35) and the controls (mean = 19.48, s.d. = 0.89) recognized these actions accurately. In the IDs, only ID2 failed to recognize one action (playing golf). This suggested that the upper-limb actions used in Experiment 2 were not difficult to recognize when presented in a more familiar format. Yet, to directly test the hypothesis that the IDs’ difficulty with the manual actions in Experiment 2 could be attributed to the format of the PLAs, we conducted an ANOVA with group as between-subject factor and format (plas*vs*pantomines) as within-subject factor on the data transformed by an ART procedure (Leys and Schumann, 2010). The interaction between group and format was significant (*F* (1, 33) = 15.4, *P* < 0.001, η^2^ = 0.32). The results of an additional Bayesian repeated-measure ANOVA indicated that the model including the interaction between group and effector was 128 times (Bayes factor) more likely than the model including only the main effects.

## Discussion

Perceiving the body movements of someone else activates brain areas not only in the observer’s visual perceptual system but also in his/her motor system. This finding has generated a debate about the role of motor representations and processes in recognizing others’ actions. According to a strictly perceptual view, action recognition relies on a visuo-perceptual analysis of the actor’s body shape and motion, which provides the visual description that ‘activates’ a representation of the corresponding action category stored in memory (Johansson, 1973; Gonzalez Rothi *et al.*, 1991; Giese and Poggio, 2003; Jellema and Perrett, 2004). On this view, although automatically activated, motor representations are not involved in the processes of action recognition. In contrast, ‘motor theories’ of action recognition propose that the recognition of others’ actions is supported by covert unconscious imitation of the observed body movements in the observer’s motor system (Rizzolatti *et al.*, 2001; Rizzolatti and Sinigaglia, 2010). To address this issue, we extended a previous study that examined whether individuals born without upper limbs (congenital aplasia) have difficulty in recognizing upper-limb actions—actions that they are unable to covertly imitate (Vannuscorps and Caramazza, 2016b). In line with the conclusion of that previous study, the results of the current study indicate that the IDs are able to recognize manual actions presented as pantomimes as efficiently as typically developed individuals. However, and in contrast with the conclusion of the previous study, we now find clear evidence that the IDs are significantly less accurate at recognizing PLAs of actions that they are unable to imitate than PLAs of actions that they can imitate.

These results contribute to constraining the contribution of effector-specific simulation to a narrower scope than that initially hypothesized by motor simulation theories. Previous studies had shown that individuals with brain damage or a congenital disorder could (i) recognize carefully crafted pictures and video clips of familiar actions that they could not covertly imitate (‘effector-specific motor simulation’) as rapidly as typically developed participants (Vannuscorps *et al.*, 2013, 2016) and (ii) even use fine-grained kinematic information to draw different types of inferences, such as the mental state of an actor or the most likely outcome of an action, as efficiently as typically developed participants (Vannuscorps and Caramazza, 2016b, 2017). The results of Experiment 1 additionally demonstrate that individuals who cannot covertly imitate observed postures and movements do not require more information to recognize an action. This finding cannot be explained by a lack of sensitivity of the measure or as a mere null effect. The measure is sensitive, with no ceiling or floor effect and a Bayes factor provided positive evidence in favor of the hypothesis that the IDs are at least as good as the control participants in this task.

Of course, this does not ‘imply’ that effector-specific motor simulation does not support the recognition of actions presented as pantomimes in typically developed participants. Correlated sensorimotor experience may be necessary for the development of motor contributions to action perception (e.g. Catmur and Heyes, 2019), and the IDs may have developed an atypically efficient visual system to compensate for their congenitally missing limbs. In this case, our results would have to be interpreted as useful evidence about the range of computational and neural plasticity that is possible in a system that typically relies on motor simulation. However, this alternate conclusion faces several challenges. First, it seems difficult to reconcile with evidence of efficient action recognition in patients with acquired motor disorders (Negri *et al.*, 2007; Kalénine *et al.*, 2010; Papeo *et al.*, 2010; Vannuscorps *et al.*, 2016) and with evidence that individuals with congenitally missing or paralyzed limbs not only are as efficient as control participants in recognizing actions but also engage the very same neural network (Vannuscorps *et al*., 2019) and perform tasks in a way that is qualitatively very similar (Vannuscorps and Caramazza, 2016b; Vannuscorps *et al.*, 2020). In addition, and more importantly, we are not aware of experimental evidence that would justify favoring this less parsimonious alternative conclusion. As reviewed elsewhere, the interpretation of the results from neuroimaging, behavioral, neuropsychological and transcranial magnetic stimulation (TMS) studies, which have been cited in support of motor simulation theories of perception, has been challenged (Vannuscorps and Caramazza, 2016b; Hickok, 2009; Caramazza *et al*., 2014). For instance, although there is clear evidence that action recognition may be modulated by concurrent motor tasks and TMS over the motor cortex, such evidence would be compelling only if one were to assume that TMS and concurrent motor tasks affect specifically and ‘only’ motor simulation (Hamilton *et al.*, 2004; Oberman *et al.*, 2007; Maringer *et al.*, 2011; Rychlowska *et al.*, 2014; Ipser and Cook, 2016; Paracampo *et al.*, 2017). There is clear evidence against this assumption. In addition to mobilizing the motor system itself, moving one’s limb feeds efferent copies and corollary discharges of the motor commands to the sensory and perceptual pathways involved in motor control (Wolpert *et al.*, 2003). For example, moving one’s body parts activates not only the motor system but also parts of the visual cortex involved in the perception of these body parts (Astafiev *et al.*, 2004; Dinstein *et al.*, 2007; Orlov *et al.*, 2010). Likewise, TMS applied to an area may have distant effects on other areas to which it projects (Ruff *et al.*, 2009; Siebner *et al.*, 2009; Papeo *et al.*, 2015). Thus, TMS applied to the motor system may have functional effects upon action recognition by modulating the visuo-perceptual system to which it is naturally connected to support the control of one’s movements. Although it is possible that the IDs’ ability to recognize upper-limb actions is supported by idiosyncratic computational and neural mechanisms, at this juncture experimental evidence on the ability to recognize familiar actions depicted in a familiar format is better and more parsimoniously explained without effector-specific motor simulation. In line with this conclusion, the results of functional magnetic resonance imaging studies conducted to identify the brain areas underlying core action recognition (i.e. the recognition of familiar actions presented in a familiar format) collectively suggest that it relies on visuo-perceptual (i.e. lateral occipito-temporal cortex and inferior parietal lobule) rather than motor areas of the brain (see Wurm and Caramazza, 2022 for review).

Nevertheless, there remains ample space for a role of effector-specific motor simulation in action recognition. The demasking paradigm used in Experiment 1 measures the amount of information required to recognize an action. However, performance in this task may reflect both immediate recognition and the result of other processes (e.g. decision and guessing), which may have differently contributed to the performance of the controls and of the IDs. Therefore, it remains possible that effector-specific motor simulation contributes to the ease or speed of action recognition. At odds with this possibility, two previous studies had reported individuals with acquired and congenital motor disorders who were able to recognize (name) pictures and video clips of actions that they could not imitate as accurately and as rapidly as typically developed participants (Vannuscorps *et al.*, 2013; Vannuscorps and Caramazza, 2016b). However, the main statistical tool used in these studies to test for a deficit in an individual patient, the modified *t-*test (Crawford and Howell, 1998), has a power of only ∼60% to detect a 2 s.d. deficit. Therefore, it remains possible that effector-specific motor simulation may contribute to some extent to the speed of action recognition. Another possibility is that effector-specific motor simulation contributes to decreasing the cognitive cost of action recognition, a hypothesis that, to our knowledge, has yet to be tested, or to the recognition of challenging action stimuli. The findings from the PLA naming task support this possibility: IDs 1–7 recognized upper-limb actions presented as PLAs significantly less accurately than lower-limb actions in PLAs (everything else being equal) and ID8 recognized PLAs of both upper- and lower-limb actions less accurately than control participants. This finding corroborates those of previous studies showing that patients with hemiplegia and paraplegia suffer from difficulties to recognize actions depicted as PLAs (Serino *et al.*, 2010; Arrighi *et al.*, 2011). Note, however, that the latter findings were interpreted, and have been cited so far, as evidence of a contribution of the motor system to core action recognition, that is, to action recognition ‘in general’. Our findings challenge this conclusion: the ID’s specific difficulty for upper-limb actions restricted to those presented as PLAs, and not to pantomimed actions, suggests that motor simulation may contribute only to the recognition of challenging action stimuli.

Future studies will be needed to explore whether effector-specific motor simulation also plays a role in the recognition of challenging real-life action stimuli. We chose to use PLAs in this study because of their widespread use in the field of action and body movement perception (Blake and Shiffrar, 2007) and previous evidence suggesting that PLAs were difficult to recognize for patients with different types of motor disorders (Serino *et al.*, 2010; Arrighi *et al.*, 2011). One could object that the PLAs have low ecological validity, raising the question whether our findings have any implication for the recognition of challenging stimuli in real life. Three main lines of evidence mitigate this concern, however. First, motor involvement during action recognition has been shown to increase in various types of challenging conditions, such as when offline TMS interferes with the observer’s visuo-perceptual system (Avenanti *et al.*, 2013), when the stimuli are meaningless (Hétu *et al.*, 2011) or when they are less familiar (Stapel *et al.*, 2010). Second, and more convincingly, the effect of various methods that interfere with motor simulation on action recognition (such as TMS and transcranial random noise stimulation applied on the observer’s premotor cortex) has been shown to be larger (Paracampo *et al.*, 2018) or even specific (Penton *et al.*, 2017; Yang and Banissy, 2017) to individuals for whom the task is most challenging (i.e. with low baseline performance). Third, although we have previously shown that the IDs perform at a typical level of efficiency in the hand laterality judgment task with simple hand postures (Vannuscorps *et al.*, 2012; Vannuscorps and Caramazza, 2015), a recent study found atypical performance in individuals born with congenital unilateral hand dysplasia in laterality judgments using more complex hand postures involving atypical finger and wrist orientations (Maimon-Mor *et al.*, 2020). For all these reasons, it is likely that motor simulation helps action recognition in a variety of challenging conditions in real life. As a first approximation, we hypothesize that motor simulation might contribute to action recognition whenever core action recognition fails. Potential reasons for core action recognition to fail include, but are not limited to, limitations linked to the observer (e.g. low visual acuity and familiarity with some actions) and information loss at the level of the stimulus itself such as when a stimulus/an action is occluded, insufficiently informative, or too unfamiliar to be automatically matched onto an action representation stored in memory. A different level of familiarity with the actions depicted as PLAs may explain, for instance, the large variability of performance in the IDs.

In sum, our findings are consistent with previously apparently diverging results, cited either in favor or against the direct-matching hypothesis of action recognition, and in fact reconcile them. Thus, they are consistent both with the finding that at least some patients with apraxia, despite their action production deficit, perform within the normal range in identifying actions from pantomimes or pictures and with the finding that patients with hemiplegia or paraplegia have difficulties in naming or detecting PLAs of actions. This emphasizes the need to reframe the current controversy regarding the role of effector-specific motor simulation in action recognition: instead of focusing on the dichotomy between motor and non-motor theories, the field would benefit from new hypotheses specifying when and how effector-specific motor simulation may contribute to the recognition of actions. Beyond this question, our findings encourage future theoretical work and empirical studies to consider with more attention the nature of the stimuli used to study the cognitive and neural bases of action recognition. Eventually, the goal should be not only to develop models detailing the cognitive and neural mechanisms underlying core action recognition but also to describe what, when and how effector-specific motor simulation may supplement core action recognition processes to accommodate the full variety of action stimuli that humans can recognize.

As a first attempt in that direction, we propose to distinguish three different routes to action recognition, schematized in Figure 1. Of course, this hypothesis is at an early stage of development, in several respects, and future studies will be needed to evaluate and refine this proposal. However, we believe that this proposal has some value in that it provides a much-needed tractable framework for thinking about when and how effector-specific motor simulation may contribute to action recognition. In this spirit, we wish to conclude by making explicit that we assume that the contribution of these different routes to action recognition depends on their availability and their efficiency. The role of availability is trivial: when one or several routes are unavailable, for instance because the stimulus is unfamiliar, or because the observer is deprived of the motor representations required to covertly imitate the observed body movements, then action recognition depends on the other route(s). The efficiency claim proposes that when several routes are simultaneously available, action recognition efficiency depends uniquely on the most efficient of these routes. The availability assumption is motivated by the IDs’ specific deficit for the upper-limb actions (that they are not able to imitate) presented as PLAs (that are not depicted in a familiar format). In turn, the efficiency assumption seems necessary to explain the IDs’ typically efficient recognition of pantomimes, pictures and video clips of upper-limb actions reported in this and previous studies.

## Data Availability

The data underlying this article are displayed in the article (Figure 2). They will be promptly shared by the corresponding author upon request.
